# Efficacy of commercial recombinant HVT vaccines against a North American clade 2.3.4.4b H5N1 highly pathogenic avian influenza virus in chickens

**DOI:** 10.1371/journal.pone.0307100

**Published:** 2024-07-16

**Authors:** Jiho Lee, Chang-Won Lee, David L. Suarez, Scott A. Lee, Taejoong Kim, Erica Spackman

**Affiliations:** 1 U.S. Department of Agriculture, Exotic and Emerging Avian Viral Diseases Unit, U.S. National Poultry Research Center, Agricultural Research Service, Athens, Georgia, United States of America; 2 U.S. Department of Agriculture, Endemic Poultry Viral Diseases Unit, U.S. National Poultry Research Center, Agricultural Research Service, Athens, Georgia, United States of America; : Keele University School of Life Sciences, UNITED KINGDOM OF GREAT BRITAIN AND NORTHERN IRELAND

## Abstract

The outbreak of clade 2.3.4.4b H5 highly pathogenic avian influenza (HPAI) in North America that started in 2021 has increased interest in applying vaccination as a strategy to help control and prevent the disease in poultry. Two commercially available vaccines based on the recombinant herpes virus of turkeys (rHVT) vector were tested against a recent North American clade 2.3.4.4b H5 HPAI virus isolate: A/turkey/Indiana/22-003707-003/2022 H5N1 in specific pathogen free white leghorn (WL) chickens and commercial broiler chickens. One rHVT-H5 vaccine encodes a hemagglutinin (HA) gene designed by the computationally optimized broadly reactive antigen method (COBRA-HVT vaccine). The other encodes an HA gene of a clade 2.2 virus (2.2-HVT vaccine). There was 100% survival of both chicken types COBRA-HVT vaccinated groups and in the 2.2-HVT vaccinated groups there was 94.8% and 90% survival of the WL and broilers respectively. Compared to the 2.2-HVT vaccinated groups, WL in the COBRA-HVT vaccinated group shed significantly lower mean viral titers by the cloacal route and broilers shed significantly lower titers by the oropharyngeal route than broilers. Virus titers detected in oral and cloacal swabs were otherwise similar among both vaccine groups and chicken types. To assess antibody-based tests to identify birds that have been infected after vaccination (DIVA-VI), sera collected after the challenge were tested with enzyme-linked lectin assay-neuraminidase inhibition (ELLA-NI) for N1 neuraminidase antibody detection and by commercial ELISA for detection of antibodies to the NP protein. As early as 7 days post challenge (DPC) 100% of the chickens were positive by ELLA-NI. ELISA was less sensitive with a maximum of 75% positive at 10DPC in broilers vaccinated with 2.2-HVT. Both vaccines provided protection from challenge to both types of chickens and ELLA-NI was sensitive at identifying antibodies to the challenge virus therefore should be evaluated further for DIVA-VI.

## Introduction

Since the emergence of A/goose/Guangdong/1/1996 (gs/GD/96) H5N1 highly pathogenic avian influenza (HPAI) virus, the lineage diversified into multiple genetic groups (clade 0–9) and multiple subclades [1,2]. Diversification of the hemagglutinin (HA) gene lead to the emergence of clade 2.3.4.4b H5, which became predominant in Asia, Africa, Europe, and the Middle East, causing devastating losses to the poultry industry since it was first identified in 2014 [1–3]. In late 2021, clade 2.3.4.4b H5N1 HPAI was detected in wild birds in North America, followed by numerous outbreaks across the continent in commercial and backyard poultry [4]. Three hundred and twenty-five commercial flocks and 507 backyard flocks were confirmed positive for the virus, affecting approximately 59 million birds in 47 states within the first 15 months of the outbreak in the US [5].

Vaccines may be utilized to help control disease and virus spread among poultry and may help prevent spill-over to other species. Historically, vaccination against HPAI has mainly been used in countries where the virus is enzootic such as Mexico, China, Egypt, Hong Kong, Indonesia, and Vietnam. Due to the on-going and widespread nature of the current clade 2.3.4.4b H5 HPAI virus outbreaks, there has been growing interest in using vaccine for the control of HPAI in poultry in other regions, for example France has begun vaccination of mule ducks in the autumn of 2023 [6].

While inactivated vaccine accounts for most of the doses of AIV vaccine used [7], live recombinant vectored vaccines such as herpes virus of turkeys (rHVT) have also been developed for HPAI. The rHVT vector has been shown to be safe and effective for numerous poultry viruses over several decades [8] and rHVT vectored vaccines for H5 HPAI have been developed and licensed in several countries. The rHVT vectored vaccines offer several advantages including the ability to replicate persistently in the host so may provide a good duration of immunity, they induce cell-mediated immunity (CMI), they can work in the presence of maternally derived antibody to the target antigen, and can be administered efficiently *in ovo* or at day of age in the hatchery [8–12]. Previous *in vivo* studies using rHVT vaccines encoding an H5 HA have demonstrated their efficacy in reducing mortality and viral shedding against other lineages of H5 HPAI viruses in poultry, including earlier clade 2.3.4.4 isolates [9,13–20]. A potential drawback is that rHVT vectored vaccines have a limited host range and are not recommended for ducks and geese [13,21,22], and can only be given one time early in life before the birds are exposed to Marek’s disease.

One of the major concerns for the implementation of HPAI vaccination programs is the ability to differentiate animals that have been infected after vaccination from animals that have been vaccinated but never infected (DIVA-VI). The primary approach is to attempt to detect antibodies to immunogenic proteins only expressed during infection: the nucleoprotein (NP) and neuraminidase (NA). Because inactivated HPAI vaccines use whole virus as the antigen and they can induce antibodies similar to infection, including the NP and homologous NA, DIVA-VI tests that target these antibodies cannot be utilized with birds vaccinated with inactivated vaccines [23]. In contrast, vectored vaccines only induce antibody to the hemagglutinin (HA), so it is simpler to apply DIVA-VI strategies. Several serological diagnostic tools routinely used for avian influenza surveillance such as ELISA, and agar gel immuno-diffusion, or immune-fluorescent assay can be used to conduct DIVA-VI surveillance [23–25], but have not been validated for this purpose. An additional assay, enzyme-linked lectin assay-neuraminidase inhibition (ELLA-NI) detects NA specific antibody so needs to be matched to the field virus, but the NA is highly immunogenic compared to the NP so has the potential to be more sensitive.

The objective of this study was to evaluate two US licensed H5 rHVT vaccines against challenge with a recent North American clade 2.3.4.4b H5N1 HPAI virus in both commercial broiler meat-type chickens and specific pathogen free (SPF) white leghorn (WL) egg layer-type chickens. One vaccine, “COBRA-HVT”, is a product that encodes an HA produced by computationally optimized broadly reactive antigen (COBRA) design, which is expected to elicit a broader antibody response against antigenically diverse challenge viruses [26,27]. The other vaccine, “2.2-HVT” contains the HA gene of A/mute swan/Hungary/4999/2006 clade 2.2 H5N1 (MS/HU/06) (GISAID accession for the HA EPI_ISL_177883) and has shown efficacy in protecting chickens against multiple clades of H5 HPAI virus infection [18,20]. Vaccine efficacy was characterized by comparison of morbidity, mortality, and virus shed by the oropharyngeal (OP) and cloacal (CL) routes, with sham vaccinated control chickens of each type. Finally, serum collected from the vaccinated chickens post challenge was used to test ELLA-NI and a commercial ELISA for anti-NP antibody for sensitivity as potential DIVA-VI tests.

## Materials and methods

### Viruses

All procedures using infectious material were reviewed and approved by the Institutional Biosafety Committee of US National Poultry Research Center (USNPRC), US Department of Agriculture-Agricultural Research Service, Athens, GA. The HPAI virus isolate A/turkey/Indiana/22-003707-003/2022 H5N1 (TK/IN/22) (GISAID accession for the HA EPI_ISL_18132254) was provided by Dr. Mia Torchetti, National Veterinary Services Laboratories, US Department of Agriculture-Animal and Plant Health Inspection Service, Ames, IA. The A/Vietnam/1203/2004 H5N1 HPAI virus (Viet/04), A/Whooper Swan/Mongolia/244/2005 H5N1 (WS/Mongolia/05) HPAI virus, and A/Flycatcher/CA/14875-1/1994 H7N1 low pathogenic avian influenza virus isolates were provided by the repository at the USNPRC. Virus isolates were propagated and titrated in SPF embryonating chicken eggs using standard procedures [28]. Titers were determined using the Reed-Muench method [29].

### Vaccines

Two commercial rHVT-H5 vaccines were selected because they are licensed in the US (and may be licensed elsewhere) and were supplied by the manufacturers: 2.2-HVT (Vectormune HVT AIV, Ceva Animal Health LLC, Lenexa, KS) (serial 395–134); and COBRA-HVT (Vaxxitek HVT+IBD+H5, Boehringer-Ingelheim Animal Health USA, Ridgefield, CT) (serial EW003) [30]. The amino acid similarity between the vaccine antigens and the challenge virus HA was 91.7% (COBRA-HVT) and 91.2% (2.2-HVT).

### Challenge study design

All animal work was reviewed and approved by the USNPRC Institutional Animal Care and Use Committee. Mixed sex, SPF WL chickens (*Gallus gallus domesticus*) were obtained at hatch from in-house flocks. Broiler chicken eggs were obtained from a commercial hatchery at 18 days of incubation prior to administration of any *in ovo* vaccines and were hatched on-site. All birds were randomly assigned to vaccine groups based on type (different genetic lines). Vaccine groups are shown in Table 1. All vaccines were prepared and administered on the day of hatch by the subcutaneous route at the nape of the neck in accordance with the manufacturer’s instructions (0.2ml per chicken). Serum was collected from all chickens 25 days post vaccination to evaluate the antibody response to the vaccines.

**Table 1:** Vaccine groups, morbidity, mortality and mean viral shedding from the oropharyngeal (OP) and cloacal (CL) routes.

Type	Vaccine	Group size	Morbidity^a^ (%positive)	Mortality^b^ (%positive)	Log_10_ per ml mean virus titer equivalent ± standard deviation (% positive)					
					2 DPC		4 DPC		7 DPC	
					OP	CL	OP	CL	OP	CL
SPF white leghorn	Sham (diluent)	20	20/20 (100)	20/20 (100)	5.6±0.4^**c**^ (100)	5.0±0.5^**c**^ (100)	N/A^e^	N/A	N/A	N/A
	COBRA-HVT	20	1/20 (5)	0/20 (0)	2.4±1.1 (90)	<0.3^f^ (0)	2.8±1.1 (95)	0.4 (5)	0.6±0.6 (25)	0.5 (5)
	2.2-HVT	19	1/19 (5.2)	1/19 (5.2)	2.8±1.1 (94.7)	0.5±0.7 (10.5)	0.8±1.2 (15.8)	2.9±1.2 (89.5)	0.9±0.9^d^ (50)	1.1±1.5^**d**^ (22.2)
Broiler	Sham (diluent)	10	10/10 (100)	10/10 (100)	5.0±0.6^**c**^ (100)	4.5±0.3^**c**^ (100)	N/A	N/A	N/A	N/A
	COBRA-HVT	10	1/10 (10)	0/10 (0)	2.0±1.6 (70)	<0.3 (0)	2.1±1.4 (70)	<0.3 (0)	1.0±0.8 (50)	<0.3 (0)
	2.2-HVT	10	1/10 (10)	1/10 (10)	2.8±1.6 (80)	<0.3 (0)	3.2±1.6 (80)	0.6 (10)	2.5±1.7 (80)	0.7 (20)

^a^no. with clinical disease/total no. of birds.

^b^no. dead/total no. of birds.

^c^Post-mortem shed data from sham vaccinated chickens that died 1day post challenge (DPC) are included in the 2DPC for the respective group of sham vaccinates (white leghorns or broilers).

^d^Post-mortem shed data from one white leghorn chicken that was euthanized 6DPC is included in the 7DPC of that group.

^e^N/A = Not applicable.

^f^Swab titers below the qRRT-PCR limit of detection (LOD) which was 0.6log_10_ 50% egg infectious dose equivalents per ml (EID_50_/ml) were assigned a value of 50% of the limit of detection (0.3log_10_ EID_50_).

Four weeks post vaccination (four weeks of age) chickens were challenged with a target dose 6.0log_10_ 50% egg infectious doses (EID_50_) per bird of TK/IN/22 in 0.1ml by the intrachoanal route (i.e., inoculation into the avian cleft palate which simulates respiratory and oral exposure and maintaining a uniform dose is easier than when using the intranasal or intraocular routes).Titration of the challenge virus after dilution confirmed the challenge dose to be 6.7log_10_ EID_50_ per bird. Oropharyngeal and CL swabs were collected from all birds at 2-, 4-, and 7-days post challenge (DPC). Swabs were also collected from dead and euthanized birds.

To evaluate antibody-based DIVA-VI tests, serum was collected at 7-, 10- and 14DPC. Mortality and morbidity were recorded for 14DPC. Surviving birds were euthanized at 14DPC. If birds were severely lethargic (exhibiting morbidity that interferes with the ability to access feed and/or water) or presented with neurological signs, they were euthanized and were counted as mortality at the next observation time for mean death time calculations. Birds were observed a minimum of twice daily during the first week after challenge and a minimum of once daily thereafter. Euthanasia was performed by cervical dislocation in accordance with American Veterinary Medical Association guidelines.

### Quantitative rRT-PCR (qRRT-PCR)

RNA was extracted from OP and CL swabs using the MagMax magnetic bead extraction kit (Thermo Fisher Scientific, Waltham, MA) with the wash modifications as described by Das *et al*., [31]. Quantitative real-time RT-PCR directed to the influenza A M gene was conducted as described previously [32] on a QuantStudio 5 (Thermo Fisher Scientific) instrument. A standard curve was generated from a titrated stock of TK/IN/22 and was used to calculate titer equivalents using the real time PCR instrument’s software.

### Hemagglutination inhibition assay

Hemagglutination inhibition (HI) assays were run in accordance with standard procedures [33]. All pre-challenge sera collected at 25 days post vaccination were tested against the challenge virus and the closest isolates available to the vaccine antigens. The serum from the 2.2-HVT group was tested against WS/Mongolia/05 (99.3% similarity) and the serum from the COBRA-HVT group was tested against Viet/04 (98.2% similarity). Titers of eight or below were considered negative. For statistical analysis negative serum was assigned an imputed value of four which is 50% of the cut off value [34].

### Commercial ELISA

A commercial AIV antibody ELISA (AI Ab Test, IDEXX laboratories, Westbrook, ME) was used in accordance with the manufacturer’s instructions. Sera were tested to detect anti-NP antibodies pre-challenge (25days pos-vaccination) and at 7-, 10- and 14DPC.

### Enzyme-linked lectin assay (ELLA) for detection of neuraminidase inhibition (NI) antibody

The ELLA was performed as previously described with minor modifications [24,25]. Briefly, the NA activity of a beta-propiolactone inactivated H7N1 virus (A/Flycatcher/CA/14875-1/1994) was quantified to determine the effective concentration (EC) of antigen. The 98% EC (EC_98_) of antigen was subsequently used for the ELLA-NI assays. For ELLA-NI assay, the antigen and serum mixture was incubated overnight (approximately18hr) at 37°C and the NA activity was determined following the procedure as described in Spackman *et al*. [25]. The average background absorbance value was subtracted from the sample absorbance value then that value was divided by the average values of wells with only NA antigen. This value was multiplied by a factor of 100 to calculate the percent NA activity. The percent NI activity of individual serum samples was determined by subtracting the percent NA activity from 100%. A cut-off value for positive NI activity was determined by adding three standard deviations to the mean NI activity of pre-challenge sera (i.e., NA antibody negative sera) of each corresponding group of chickens at 7-, 10- and 14DPC. Each serum was tested at dilutions of 1:20 and 1:40.

### Statistical analysis

All statistical analysis were performed using GraphPad Prism version 10.0.2 for Windows (GraphPad Software, Boston, Massachusetts USA). Mean serum antibody HI titers between groups were compared using the Mann-Whitney test. Mean viral shedding of challenged chickens was compared using two-way ANOVA with Tukey’s multiple comparison. Samples that were not detected by rRT-PCR were given a value of 50% of the assay limit of detection [34]. Note that post-mortem shed titer data from sham vaccinated chickens that were dead or euthanized on 1DPC are merged with 2DPC data and shed data from one WL chicken in the 2.2-HVT group that was euthanized on 6DPC was merged with 7DPC data of the same group. Non-specific NI antibody titers among pre-challenge sera, and post-challenge N1 antibody titers among vaccine groups and collection time points were compared using one-way ANOVA. The Pearson coefficient correlation (two-tailed test of significance) was used to analyze ELLA-NI versus oropharyngeal viral shedding titers.

## Results

### Antibody response to the vaccines

**Fig 1:**
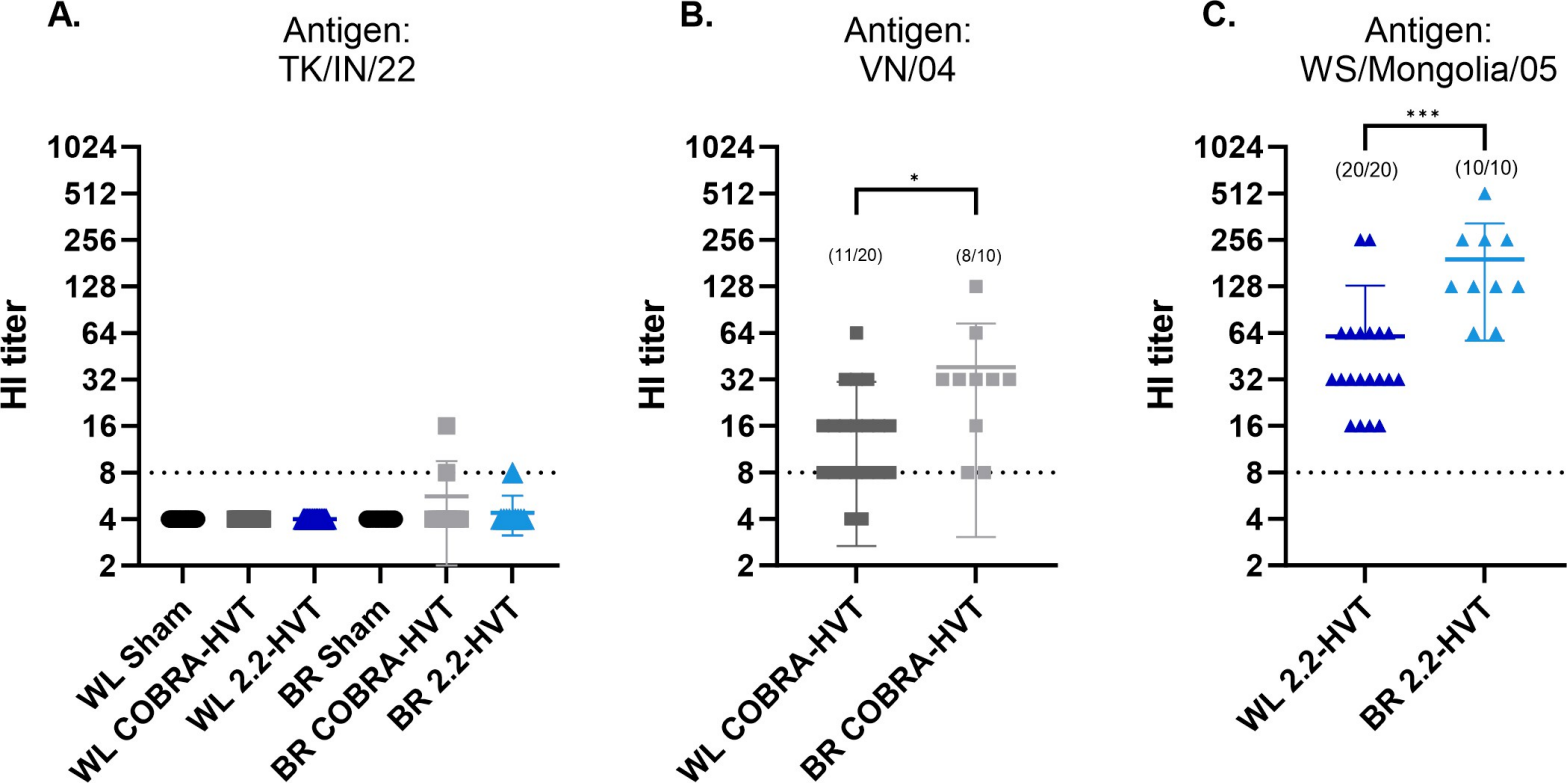
Mean antibody Hemagglutination Inhibition titers of vaccinated chickens 25 days post vaccination (pre-challenge). Titers were determined against three different antigens, TK/IN/22 (A), Viet/04 (B), and WS/Mongolia/05 (C). Titers of eight or lower are regarded as negative and sera negative by HI assay were assigned an imputed titer of four, which is 50% of the cut off value. P values are represented as * for p <0.05, and *** for p<0.001.

The pre-challenge sera from chickens were tested for reactivity against the challenge virus, TK/IN/22, and closest available isolates to each vaccine antigen. Among available isolates, the Viet/04 isolate (clade 1) was the closest to the HA of COBRA-HVT with 98.2% similarity in amino acid sequence and the WS/Mongolia/05 (clade 2.2) was the closest isolate available to the HA of 2.2-HVT, with 99.3% similarity in amino acid sequence. Only one broiler in the COBRA-HVT vaccinated group elicited an HI antibody titer above eight to TK/IN/22 (Fig 1A). When using isolates related to the vaccines, broilers vaccinated with COBRA-HVT had a geometric mean titer (GMT) of 35.3, which was significantly higher than WL chickens with the same vaccine (GMT = 16.8) (p = 0.0229). The broilers also had a higher rate of seroconversion at 80% (8/10) compared to the WL with 55% (11/20) (Fig 1B). Likewise, broilers vaccinated with 2.2-HVT had a significantly higher GMT (192.0) when compared to WL (60.8) (p = 0.0001), and 100% of both WL and broiler chickens were positive for HI antibody to the WS/Mongolia/05 isolate (Fig 1C).

### Survival rate and clinical signs

**Fig 2:**
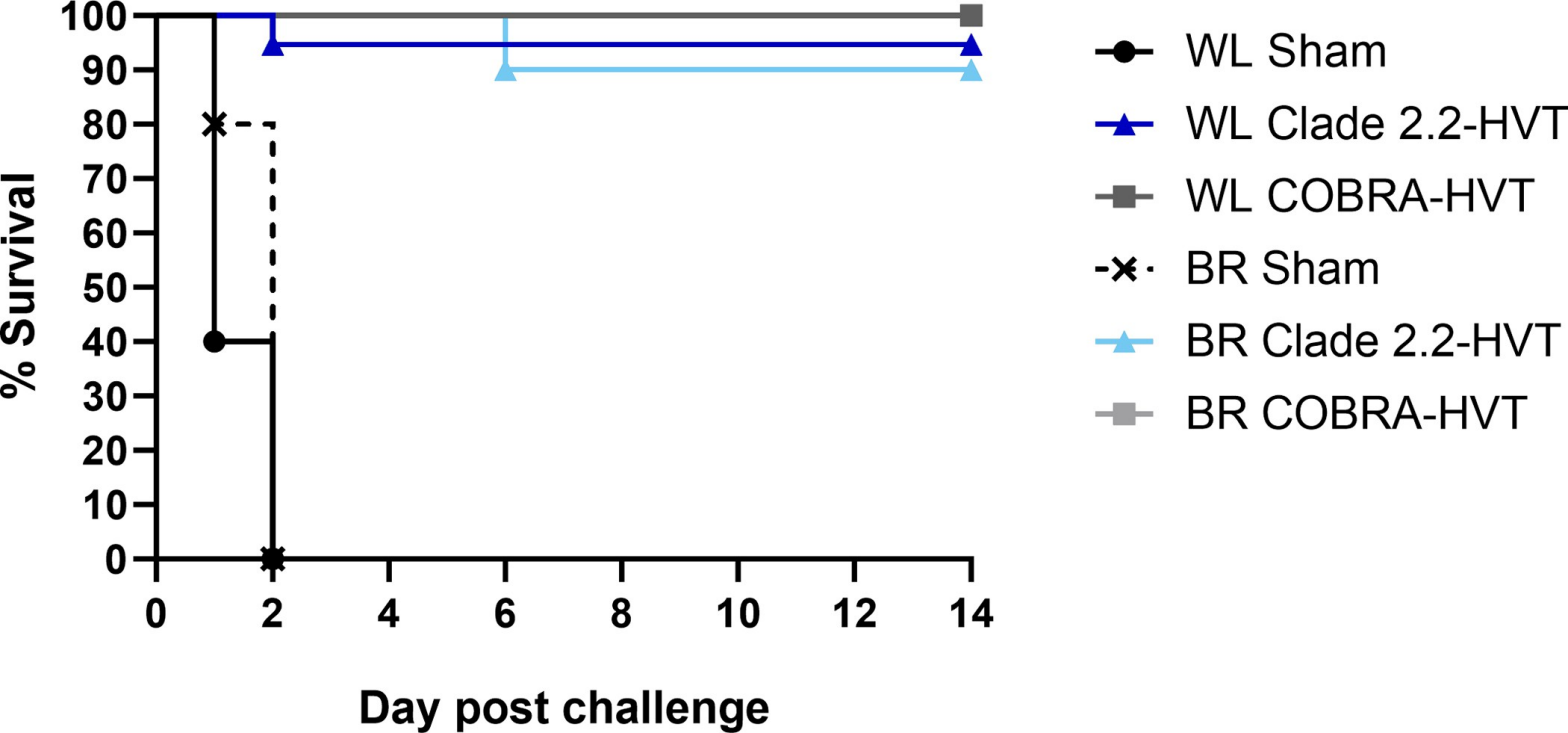
Survival curve of chickens vaccinated with sham vaccine, COBRA-HVT, or 2.2-HVT after challenge with TK/IN/22 HPAI virus.

All chickens in sham vaccinated groups died or were euthanized before or at 2DPC, with a mean death time of 1.4 days for WL and 1.8 days for broilers (Fig 2). Birds in both sham vaccinated groups presented with severe lethargy, infraorbital swelling, or sudden death. There was no mortality in the COBRA-HVT vaccine groups and clinical signs were only observed in one WL with mild lethargy and one broiler with diarrhea. In the 2.2-HVT vaccinated groups, one WL chicken and one broiler were euthanized due to severe lethargy on 4DPC and 6DPC, respectively (survival rate of 94.7% for the WL chickens and 90% for the broilers).

### Viral shedding

**Fig 3:**
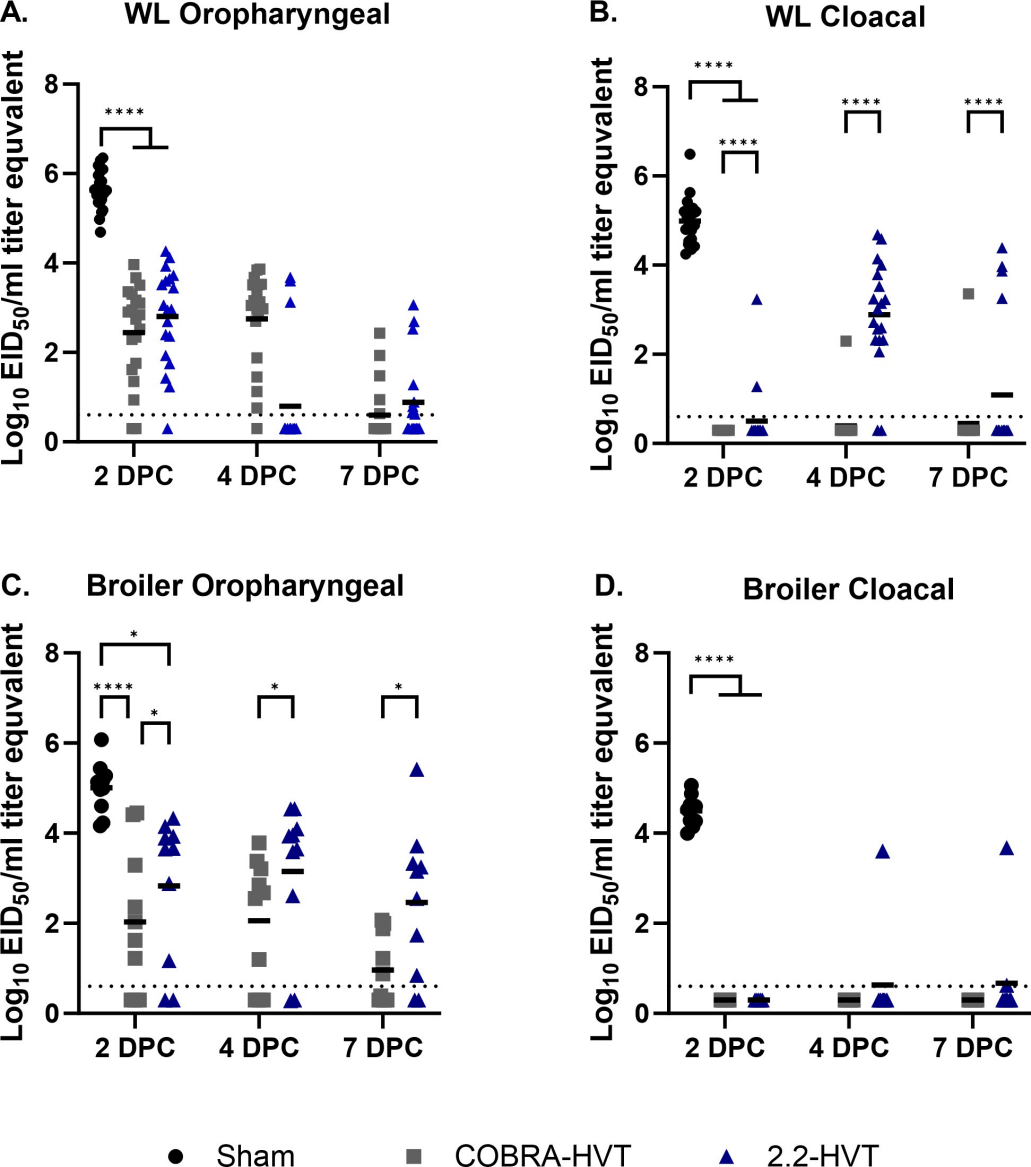
Viral shedding titer equivalents from oropharyngeal and cloacal swabs collected from white leghorn chickens (A and B) or broiler chickens (C and D) 2-, 4- and 7-days post challenge (DPC). Titers were measured using quantitative rRT-PCR and converted into log_10_ 50% egg infectious doses (EID_50_) per ml titer equivalents using the standard curve with RNA from quantified virus. Post-mortem shed titer data from sham vaccinated chickens that were dead or euthanized on 1DPC are merged with 2DPC and shed data from one WL chicken in the 2.2-HVT group that was euthanized on 6DPC is merged with 7DPC data of the same group. Columns for the sham vaccinated birds are not shown for 4 and 7 days because there were no birds left in these groups at those time points. P values are represented as * for p <0.05, and **** for p value <0.0001.

Oropharyngeal and CL viral shedding at 2-, 4-, and 7DPC was quantified by qRRT-PCR, which may not be the same as infectious virus. All chickens in the sham vaccinated groups were positive for both OP and CL viral shedding and shed significantly higher titers from both routes when compared to rHVT vaccinated chickens at 2DPC (Fig 3). For WL chickens vaccinated with COBRA-HVT, the proportion that shed detectable levels of virus by the OP or CL route at least once during the course of the study was 95.0% (19/20) and 94.7% (18/19) of the 2.2-HVT vaccinated WL chickens. There was no significant difference in virus shed quantities from the OP route by WL chickens was observed between the two vaccine groups. However, shedding by the CL route was significantly lower by the COBRA-HVT vaccinated WL chickens than the 2.2-HVT vaccinated WL chickens (p<0.0001).

Of the broilers vaccinated with COBRA-HVT 80.0% (8/10) shed detectable levels of virus at least once by the OP or CL route and those vaccinated with the 2.2-HVT vaccine had a shed detection rate of 90.0% (9/10). Significant differences between the two vaccine groups were observed in titers from OP route, where the 2.2-HVT group shed higher mean titers than the COBRA-HVT vaccinated group (p<0.05) at all three times points.

### Detection of N1-specific antibody from post-challenge sera by ELLA-NI

**Fig 4:**
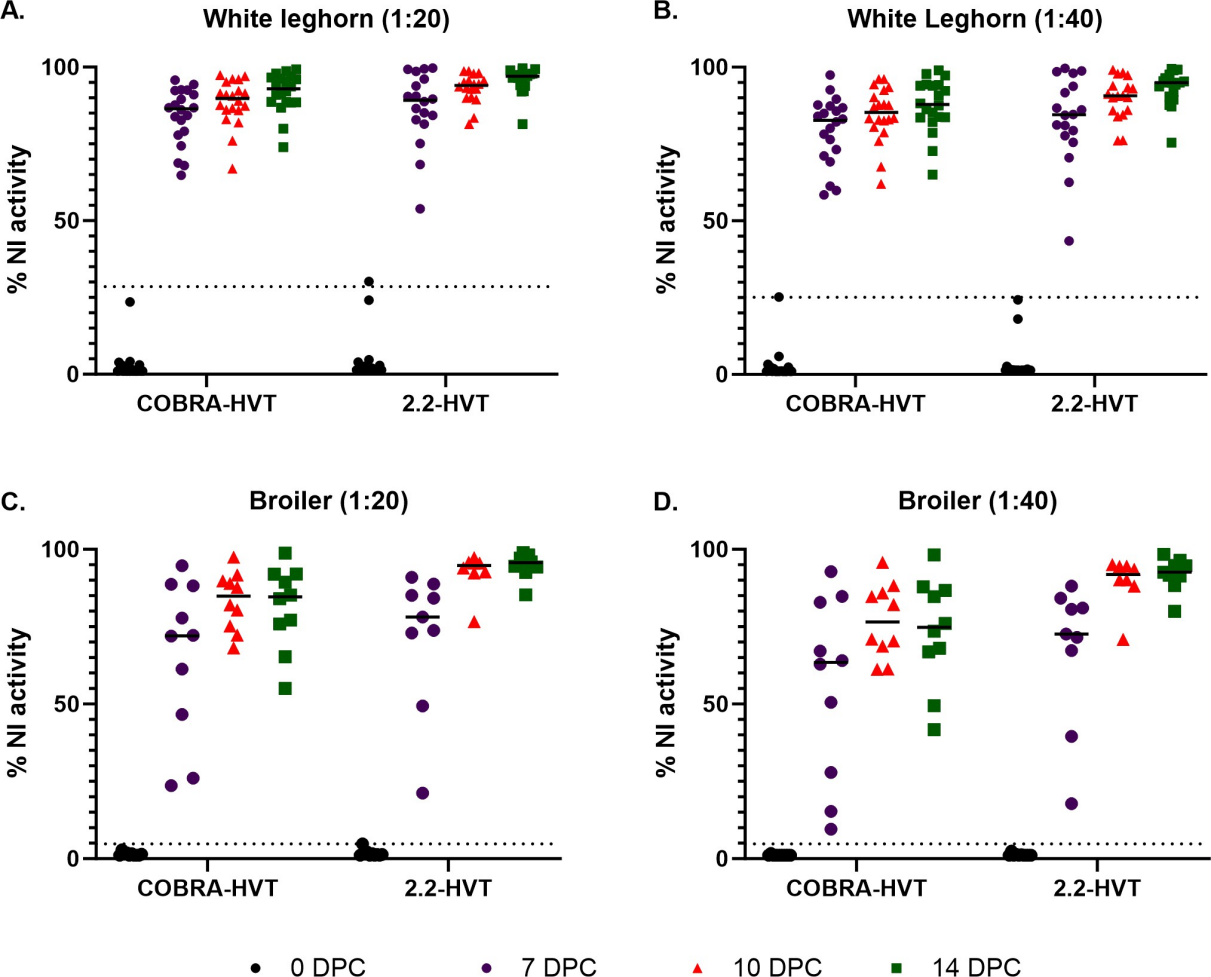
Antibody to the N1 NA in post-challenge sera of chickens, measured by percent inhibition of neuraminidase activity by enzyme linked lectin assay-neuraminidase inhibition. Post-challenge sera were collected 7-, 10-, and 14-days post challenge (DPC). (A) White leghorns with serum at a 1:20 dilution; (B) White leghorns with serum at a 1:40 dilution; (C) Broilers with serum at a 1:20 dilution; (D) Broilers with serum at a 1:40 dilution. Horizontal dotted lines indicate the cut-off determined by adding three standard deviations to the mean NI activity of pre-challenge sera from each group. Solid horizontal lines in each group indicate the median.

ELLA-N1 was used to detect N1-specific antibody induced by infection with the H5N1 challenge virus. To characterize background (non-specific inhibition of neuraminidase activity) pre-challenge sera from sham vaccinates and each vaccine groups were evaluated (S1 Fig). Overall, nonspecific NI antibody activity was around 5% in broilers and WL chickens and was not statistically different (P = 0.92). However, pre-challenge sera from three WL chickens produced non-specific reactivity which was greater than 15% at both the 1:20 and 1:40 dilutions (these were negative by ELISA). The mean percent inhibition value plus three standard deviations was calculated from the pre-challenge sera for WL and broiler chickens separately and was used to determine the cut-off to evaluate N1 specific antibody detection in post-challenge sera. At 7-, 10- and 14DPC antibodies to the N1 were detected in serum from 100% of the challenged birds regardless of the vaccine group or chicken type (Table 2 and Fig 4). At a 1:20 dilution of sera, the NI value was greater than 50% in sera from all WL chickens at all time points and at 10 and 14DPC from broiler chickens. Neuraminidase inhibition activity increased, and inhibition variability decreased in sera collected at the later time points (10 and 14DPC) compared with 7DPC although statistically significant differences were only observed between 7 and 14DPC samples (WL with 2.2-HVT (1:20) P = 0.0102 (1:20); WL with COBRA-HVT (1:20) P = 0.0246; and WL with 2.2-HVT (1:40) P = 0.0262). Results with serum at 1:40 were qualitatively similar to those at 1:20. The difference in the level of NI antibody response observed by the vaccine groups also correlated moderately with the virus shed titers (S1 Table). One sample in the 10DPC of 2.2-HVT broiler group was insufficient to perform serologic tests.

**Table 2:** Antibody to the N1 NA and NP proteins in serum collected at 7-, 10- and 14-days post challenge (DPC) from chickens vaccinated with rHVT H5 avian influenza vaccines determined by ELLA-NI (serum 1:20 and 1:40 yielded the same results) and ELISA.

Vaccine	Breed	ELLA-NI			ELISA (NP)			
		7 DPC ^a^	10 DPC	14 DPC	7 DPC	10 DPC	14 DPC	Cumulative^b^
COBRA-HVT	White leghorn	100 (20/20)	100 (20/20)	100 (20/20)	5 (1/20)	25 (5/20)	25 (5/20)	25 (5/20)
	Broiler	100 (10/10)	100 (10/10)	100 (20/20)	10 (1/10)	20 (2/10)	10 (1/10)	20 (1/10)
2.2-HVT	White leghorn	100 (18/18)	100 (18/18)	100 (18/18)	16.7 (3/18)	27.8 (5/18)	27.8 (5/18)	27.8 (5/18)
	Broiler	100 (9/9)	100 (8/8)	100 (9/9)	11.1 (1/9)	75 (6/8)	55.5 (5/9)	66.7 (6/9)

^a^ Percent (No. of positive birds/ no. of total birds).

^b^ Percent of birds with at least one positive result by ELISA.

### Detection of NP antibody by commercial ELISA in post-challenge serum

Serum antibody to the NP in sera collected at 7-, 10-, and 14DPC was measured with a commercial ELISA kit. Detection varied among the groups at 7DPC between 5.0% and 16.7% (Table 2). At 10DPC it increased to 25–75.0% and 25–55.5% at 14DPC. The cumulative percent positive rate of vaccinated groups was in descending order: 2.2-HVT broilers with 66.7% (6/9) positive, 2.2-HVT WL chickens with 27.8% (5/18) positive, COBRA-HVT WL chickens with 25.0% (5/20) positive, and COBRA-HVT broilers with 20.0% (1/10) (Table 2).

## Discussion

Recombinant HVT vectored vaccines have been used in commercial chickens for numerous viruses for many years and are administered either *in ovo* or at hatch [8]. In this study, two commercially available H5 rHVT vaccines were evaluated for protection against a recent North American clade 2.3.4.4b H5N1 HPAI virus. The HA antigens in both vaccines are genetically distinct from the HA of the challenge virus with 91.7% (COBRA-HVT) and 91.2% (2.2-HVT) amino acid similarity. Previous studies have shown that rHVT-AI vaccines can provide adequate protection with amino acid similarities in this range but are more likely to provide less protection than more closely matched vaccines [35] therefore testing is needed to ensure efficacy.

Vaccinated groups elicited antibody that could be detected by HI assay against a closely related isolate, but not against the challenge virus, except for one broiler in the COBRA-HVT group. The COBRA-HVT vaccinated birds had a 100% survival rate and 2.2-HVT vaccinated WL chickens and broilers had 94.8% and 90.0% survival rates respectively, which are higher than the efficacy requirement of 80% proposed by the World Organization for Animal Health [36]. Viral shedding by vaccinated groups was significantly reduced when compared to the sham vaccinated groups. Although no significant difference between the two vaccines was observed in OP viral shedding titers by WL chickens and CL viral shedding titers by broilers, the mean viral shedding titers from CL swab samples of WL chickens and OP swabs of broilers were significantly lower in COBRA-HVT vaccinated chickens than those of the 2.2-HVT vaccinated chickens, suggesting that virus replication was reduced more in these vaccine groups. There were no clear differences between white leghorns and broilers in virus shedding. The results agree with previous reports that have shown rHVT vaccines provided protection from mortality and a reduction in viral shedding with low or undetectable antibody to a genetically divergent challenge virus [9,18,35,37]. Therefore, HI antibody titers to the challenge virus are not a good predicter of protection with rHVT vaccines. Unfortunately, the lack of simple and reliable test to predict protection by vectored poultry vaccines remains an impediment to wider use of the vaccine.

As a live vaccine, rHVT elicits CMI which contributes to protection, but it is difficult to quantify in poultry due to a lack of simple tests. Several studies have characterized the CMI elicited by HVT in immunological studies. Heller *et al*. reported an increase in natural killer cell activity induced by the rHVT vaccine [38]. Rauw *et al*. demonstrated that *in ovo* vaccination with rHVT against Newcastle disease virus followed by *ex vivo* antigenic stimulation induced the production of IFN-ɣ in splenocytes and peripheral blood lymphocytes [39]. In a study performed by Kapczynski *et al*., cross reactive cytotoxic T lymphocyte activity against H5, H6, H7, and H9 HA proteins was observed in the splenic T lymphocyte of chickens vaccinated with 2.2-HVT [9]. It appears that the combined CMI/antibody response can tolerate greater variation between the challenge virus and vaccine antigen and still allow protection [35].

The duration of Immunity with rHV” vac’ines is likely an advantage, but it needs to be characterized better for HPAI vaccines. The rHVT vector cause persistent infections that can potentially elicit continued antigenic stimulation resulting in a long-term immune response. One study with an rHVT Newcastle disease virus vaccine showed protection from virulent challenge for 72 weeks after a single vaccination [40]. Although there are many similarities between HPAI and Newcastle disease, these data need to be confirmed for HPAI because of two differences between the viruses that might reduce protection; 1) HPAI has a much shorter MDT; and 2) HPAI has a much greater capacity for antigenic variability. An additional consideration is that earlier studies with rHVT HPAI vaccines have shown clear benefits from a prime-boost approach because of better antibody responses, which have positive predictive value with improved protection [19,41]. It remains likely that multiple doses of vaccine may be needed in the real-world to maintain adequate protection in long-lived poultry like chicken breeders, layer chickens, or turkey breeders.

In addition to protection from clinical disease, reduction of transmission is an important result of vaccinating for HPAI. This could not be directly tested in our vaccine trials but has been shown to be successful in previous studies with similar vaccines and challenges with clade 2.3.4.4b H5 HPAI virus isolates. Germeraad *et al*., demonstrated that these same two rHVT vaccines did reduce transmission of a 2021 European clade 2.3.4.4b H5 HPAI virus in chickens [42]. A study by Palya *et al*. also demonstrated reduced transmission among chickens by the 2.2-HVT vaccine against a 2017 European clade 2.3.4.4b H5 HPAI virus challenge [20]. Therefore, rHVT vaccines have been shown to effectively reduce HPAI transmission in experimental settings.

Currently a major impediment to the use of vaccination is the negative effects it could have on the trade of poultry and poultry products. A reliable and cost-effective DIVA-VI surveillance test to demonstrate that vaccinated poultry were not infected with field virus would help ameliorate concerns about vaccines possibly masking infection. This may be accomplished with an assay for antibody that is only elicited after infection [23] but there is limited field data. Also, tests must be matched with specific vaccines and field viruses so developing widely applicable kits is difficult. Because rHVT vaccines are subunit vaccines that only express the HA protein, two targets are available, the NP and the NA protein. The advantage of looking for antibody to the NP is that currently licensed commercial ELISA kits can be used. ELISA kits for NA detection are not widely available and have not been developed for all NA subtypes, which need to be antigenically matched to the field virus. Therefore, an alternative NA antibody assay which can accommodate rapid changes of the antigen, such as the ELLA-NI used here, is an option.

Using ELLA-NI, we were able to detect N1 antibodies in 100% of the serum samples regardless of vaccine, time post challenge, or chicken type. Similarly, a previous report showed that ELLA-NI could effectively detect N1 specific antibodies in sera from chickens vaccinated with an inactivated H5N9 vaccine or an H5-RNA particle vaccine then challenged with a clade 2.3.4.4b H5N1 HPAI virus [25]. More data are needed for validation of the ELLA-NI and specificity needs to be shown with field serum to define the optimal cut-off value. For the current and previous experimental vaccine studies a cut-off of the mean plus three standard deviations was used and more conservative alternative cut-off values of 25% and 50% reduction in NA activity were also evaluated and gave similar results [25]. However, field reared birds will have been exposed to more vaccines and other antigen sources, which increases the potential for non-specific reactions.

In contrast to the ELLA-NI, the cumulative sensitivity for the commercial ELISA was around 25%. Importantly, these kits have not been optimized or validated for DIVA-VI use. Although the ELLA-NI has higher sensitivity, ELISA kits may be practical in certain situations as long as the sample size accounts for the difference in sensitivity to achieve the desired target detection level.

An additional important aspect of applying any DIVA-VI test is that as vaccinal protection increases viral replication is reduced more. Therefore, the antibody response of well vaccinated birds to the field virus will be lower and will require a more sensitive DIVA-VI testing approach. Here this was shown as a moderate correlation between the ELLA-NI and oropharyngeal virus shedding.

This study demonstrated the efficacy of two commercially available rHVT-H5 vaccines against a recent clade 2.3.4.4b H5N1 HPAI virus isolate from North America. Although there was antigenic distance between vaccine antigens and the challenge strain, and low level of humoral antibody was elicited to the challenge strain by the vaccines, a significant reduction in morbidity, mortality, and viral shedding was observed. Because rHVT-AI vaccines are more robust to antigenic variation than inactivated vaccines for HPAI [35], these vaccines should be able to protect against some degree of future variability as the virus evolves. While commercial ELISA used in this study showed limited sensitivity in detecting infected birds, ELLA-NI showed high sensitivity for post-challenge DIVA-VI, which correlated with the level of virus replication as measured by virus shedding. Further studies to evaluate how the rHVT vaccines affect transmission as well as prime-boost experiments should be performed to understand the efficacy of rHVT-AI more fully in the field.

## Supporting information

S1 Fig Percent inhibition of N1 NA activity by pre-challenge sera collected 25 days post vaccination determined by enzyme linked lectin assay-neuraminidase inhibition to evaluate non-specific reactivity. (TIF)

S1 Table Correlation between ELLA-NI assay (1:20 and 1:40 serum dilutions) and oropharyngeal viral shedding by chickens. The highest oropharyngeal viral shedding titer by each bird observed during the course of experiment was selected to approximate the extent of virus replication and was compared with the percent NA inhibition by serum from the same chicken by ELLA-NI assay. Chickens that died or were euthanized have been excluded. CI = confidence interval.
(DOCX)
