# Supplementary material for: Efficacy of commercial recombinant HVT vaccines against a North American clade 2.3.4.4b H5N1 highly pathogenic avian influenza virus in chickens
Source: PLoS One. 2024 Jul 16;19(7):e0307100. doi: 10.1371/journal.pone.0307100 (PMC11251577; doi:10.1371/journal.pone.0307100)
Supplement: S1 Table — The highest oropharyngeal viral shedding titer by each bird observed during the course of experiment was selected to approximate the extent of virus replication and was compared with the percent NA inhibition by serum from the same chicken by ELLA-NI assay. Chickens that died or were euthanized have been excluded. CI = confidence interval. (DOCX) [file pone.0307100.s002.docx]

**S1 Table.** Correlation between ELLA-NI assay (1:20 and 1:40 serum dilutions) and oropharyngeal viral shedding by chickens. The highest oropharyngeal viral shedding titer by each bird observed during the course of experiment was selected to approximate the extent of virus replication and was compared with the percent NA inhibition by serum from the same chicken by ELLA-NI assay. Chickens that died or were euthanized have been excluded. CI=confidence interval.

|  | **ELLA (1:20)** | | |  | **ELLA (1:40)** | | |
| --- | --- | --- | --- | --- | --- | --- | --- |
|  | **7 DPC** | **10 DPC** | **14 DPC** |  | **7 DPC** | **10 DPC** | **14 DPC** |
| Pearson R  (95% CI) | 0.4331  (0.1944-0.6233) | 0.4218  (0.1787-0.6164) | 0.4776  (0.2479-0.6565) |  | 0.4472  (0.2112-0.6339) | 0.4311  (0.1897-0.6234) | 0.4905  (0.2636-0.6660) |
| P value | 0.0008 | 0.0012 | 0.0002 |  | 0.0005 | 0.0009 | 0.0001 |
